# Association of Clinical Scores and Cardiac Troponin I with 30-Day Mortality in Patients with Spontaneous Intracerebral Hemorrhage

**DOI:** 10.3390/epidemiologia7020043

**Published:** 2026-03-16

**Authors:** Nina Mihic, Ivan Cavar, Jelena Sulic, Katarina Vukojevic, Mirela Mabic, Sandra Lakicevic, Ante Kvesic

**Affiliations:** 1 Institute of Health Insurance of Herzegovina-Neretva County, 88000 Mostar, Bosnia and Herzegovina; mihic.nina77@gmail.com; 2 Department of Neurology, University Hospital Center Mostar, 88000 Mostar, Bosnia and Herzegovina; sandrajuric@gmail.com; 3 Department of Physiology and Immunology, School of Medicine, University of Mostar, 88000 Mostar, Bosnia and Herzegovina; 4 Department of Anatomy, Histology and Embryology, School of Medicine, University of Split, 21000 Split, Croatia; katarina.vukojevic@mefst.hr; 5 Faculty of Economics, University of Mostar, 88000 Mostar, Bosnia and Herzegovina; mirela.mabic@gmail.com; 6 Department of Surgery, University Hospital Center Mostar, 88000 Mostar, Bosnia and Herzegovina; antekvesic62@gmail.com

**Keywords:** spontaneous intracerebral hemorrhage, ICH score, high-sensitivity cardiac troponin I, age, Glasgow Coma Scale, hematoma volume, intraventricular hemorrhage, prognostic factors, mortality

## Abstract

Background/Objectives: Spontaneous intracerebral hemorrhage (sICH) is a particularly severe subtype of stroke, characterized by high rates of mortality and long-term disability, for which robust prognostic markers are still lacking. The aim of this study was to assess the relationship of the ICH score, the National Institutes of Health Stroke Scale (NIHSS) score, and serum high-sensitivity cardiac troponin I (hs-cTnI) levels with 30-day mortality in patients with sICH. Methods: We conducted a prospective observational cohort study enrolling 100 consecutive patients diagnosed with sICH based on neuroimaging findings. Demographic data, clinical parameters, neuroimaging findings, and serum hs-cTnI levels were collected on admission. Subsequently, the ICH score, its individual components, and the NIHSS score were assessed. Results: Patients who died were older and had significantly higher ICH and NIHSS scores, lower Glasgow Coma Scale (GCS) scores, larger hematoma volumes, more frequent intraventricular hemorrhage (IVH), and elevated hs-cTnI levels compared to survivors. Serum hs-cTnI concentrations were significantly correlated with ICH and NIHSS scores, lower GCS scores, larger hematoma volumes, and the presence of IVH. On univariate logistic regression, higher ICH score, NIHSS score, and hs-cTnI level were associated with mortality, whereas multivariate analysis identified the GCS score, hematoma volume, and IVH score as significant independent factors related to fatal outcome. Conclusions: Individual components of the ICH score may provide useful information on outcomes in patients with sICH. Higher serum hs-cTnI levels were associated with 30-day mortality but were not independent predictors. These markers may assist in patient monitoring and support established clinical procedures in therapeutic decision-making. Nevertheless, larger multicenter studies are needed to further clarify their clinical implications in sICH management.

## 1. Introduction

Hemorrhagic stroke, characterized by bleeding within the brain parenchyma with possible ventricular extension, accounts for nearly 11% of all stroke cases in high-income countries, with the proportion rising to around 22% in low- and middle-income countries [1,2,3]. It carries a markedly greater burden of morbidity and mortality than ischemic stroke, which is reflected in case fatality rates of around 40% within the first month and 54% after one year, while long-term functional independence is attained by approximately 12–39% of survivors [4]. The most common form of hemorrhagic stroke is the spontaneous, non-traumatic rupture of cerebral blood vessels, known as spontaneous intracerebral hemorrhage (sICH), which carries a poor prognosis: nearly half of patients die during the first month, and 80% of those who survive require help with daily activities [1,3].

Systemic arterial hypertension and cerebral amyloid angiopathy are the two most important and well-established risk factors for sICH [5,6]. Additional factors associated with sICH include advanced age, male sex, Asian ethnicity, excessive alcohol consumption, smoking, low plasma low-density lipoprotein cholesterol and triglyceride levels, cerebral microbleeds, chronic kidney disease, and the use of medications such as anticoagulants, antiplatelets, and sympathomimetics. Clinical manifestations of ICH vary according to the size and location of the hemorrhage and may include headache, nausea and vomiting, decreased level of consciousness, stupor or coma, seizures, higher cortical dysfunction, cranial nerve abnormalities, motor deficits, and cognitive impairment [4].

Considerable progress has been made in recent years in understanding the pathophysiology of sICH, identifying causative mechanisms, and improving acute management [7]. Although several therapeutic approaches have been established, including hemostatic agents, acute blood pressure reduction, intraventricular alteplase administration, and surgical intervention, none have been conclusively proven to improve functional recovery in individuals with sICH [8]. This highlights ongoing clinical challenges and the critical need for reliable prognostic biomarkers [7].

Prognostic factors consistently associated with poor outcomes include increased ICH and National Institutes of Health Stroke Scale (NIHSS) scores, age over 80 years, a low Glasgow Coma Scale (GCS) score at admission, a hematoma volume greater than 30 mL, delayed perihematomal edema, intraventricular hemorrhage (IVH), and infratentorial location of bleeding [4,9,10]. Comorbidities such as diabetes and renal dysfunction further worsen the prognosis in patients with sICH [11]. A post hoc analysis of a large randomized multicenter trial in China (CRRICH) identified right hemispheric involvement, IVH, advanced age, and higher NIHSS scores as factors associated with worse outcomes in managed conservatively patients [12,13]. Furthermore, unfavorable outcomes following hematoma surgery have been associated with an age >58 years, elevated plasma glucose levels, GCS ≤8, a larger hematoma volume, IVH, infratentorial hematoma location, and midline shift [14]. In this context, prognostic scoring systems such as the ICH and NIHSS have been developed to assist physicians in assessing prognosis [15,16].

The cardiac troponin (cTn) complex, comprising three regulatory proteins (cTnI, cTnT, and cTnC), plays a crucial role in regulating cardiac contractility [17]. Among them, cTnI and cTnT are cardiac-specific isoforms clinically recognized as highly sensitive and specific “gold-standard” biomarkers for acute myocardial infarction [18,19,20]. Beyond myocardial ischemia, elevated cTn levels are frequently observed in various acute conditions, including tachyarrhythmias, heart failure, pulmonary embolism, shock, sepsis, and non-cardiac surgical procedures [21]. Elevated cTnI levels are commonly observed in patients with sICH, suggesting potential prognostic significance [22,23,24]. It has been suggested that this increase in cTnI indicates acute myocardial injury influenced by the brain–heart axis rather than resulting from primary myocardial ischemia [25]. Several studies have demonstrated associations between elevated serum cTn levels and poor functional outcomes, as well as increased mortality [26,27], although others have failed to confirm such relationships [22,28]. Thus, the prognostic relevance of elevated cTn in the context of sICH remains uncertain [25].

Although several studies have investigated the prognostic value of the ICH score and its components, the NIHSS score, and serum cTnI levels in patients with sICH, the findings remain inconsistent and insufficiently comprehensive. Consequently, the objective of this study was to investigate the relationship between ICH characteristics and serum cTnI levels, as well as their association with mortality in patients with sICH.

## 2. Materials and Methods

### 2.1. Study Design and Participants

A total of 100 hospitalized patients, including 46 men and 54 women, with neuroradiologically confirmed sICH were enrolled in this prospective observational cohort study conducted between April 2024 and April 2025 at the Department of Neurology, University Hospital Center (UHC) Mostar, Bosnia and Herzegovina. Exclusion criteria were incomplete medical documentation, traumatic ICH, and secondary ICH attributable to ruptured aneurysm, vascular malformation, or hemorrhagic transformation of ischemic stroke. The mean age of participants was 73.59 ± 12.57 years (range: 38–93 years). The follow-up period lasted 30 days from hospital admission. Vital status at 30 days was ascertained for all patients through hospital medical records and, when applicable, by telephone contact with patients or their families after discharge. In-hospital deaths were recorded directly from institutional databases, while post-discharge outcomes were verified using electronic health records and follow-up calls. No patient was lost to follow-up during the 30-day observation period.

This study was conducted solely to collect data for research purposes. Prior to participation, all patients provided written informed consent confirming their understanding of the study procedures, potential risks and benefits, and overall purpose. For patients unable to provide consent owing to their medical condition, consent was obtained from their closest relatives or legal guardians. Consent forms were written in Croatian to ensure comprehensibility. All participants were made aware of their right to discontinue participation at any point without justification and were guaranteed full anonymity and protection of their data. Access to medical documentation was restricted to authorized research personnel and members of the Ethics Committee.

Ethical approval for this study was obtained from the Ethics Committee of UHC Mostar (Approval No. 02-I-1479/23). The research was carried out following the principles outlined in the Declaration of Helsinki.

### 2.2. Data Acquisition

All patients underwent neurological examination, standard laboratory testing, and neuroradiological brain imaging with multislice computed tomography using a 256-slice GE Revolution CT scanner (GE Medical Systems, Waukesha, WI, USA). The ICH score is a widely used prognostic tool for estimating 30-day mortality in patients with sICH [29]. It is calculated by summing points across five clinical and radiological variables: GCS score, ICH volume, presence of IVH, infratentorial hematoma location, and age (Table 1). Higher scores are associated with an increased risk of short-term mortality [15].

To assess level of consciousness, the GCS score evaluates three areas: eye opening (1–4 points), verbal response (1–5 points), and motor response (1–6 points), with an overall score ranging from 3 (deep coma) to 15 (fully alert). In general, a score ≤8 indicates a comatose state. The volume of intracerebral hematoma was estimated using the simplified ABC/2 method, a validated and widely applied approach in clinical practice. In this method, A corresponds to the largest hematoma diameter on an axial CT slice (cm), B is measured perpendicular to A on the same slice, and C is calculated by multiplying the count of axial slices showing hemorrhage by the slice thickness (cm). The volume of the hematoma was then computed using the formula Volume = (A × B × C)/2 and expressed in cubic centimeters (cm^3^) equivalent to milliliters (1 cm^3^ = 1 mL). A volume ≥30 mL was considered a significant risk factor and scored accordingly on the ICH scale.

The NIHSS is a validated clinical instrument for evaluating the severity of neurological deficits in patients presenting with acute stroke. It consists of 11 items evaluating consciousness, vision, motor and sensory function, coordination, speech, and language. Higher scores indicate more severe neurological impairment. In addition to its role in clinical monitoring, the NIHSS is widely recognized as a prognostic tool [16].

Peripheral venous blood samples were obtained from each participant via cubital venipuncture and analyzed at the Laboratory for Molecular Diagnostics, UHC Mostar. Quality control was maintained using internal laboratory procedures compliant with ISO 15189 accreditation standards [30]. All analyses were performed in a sex-partitioned manner based on the established upper reference limits. In addition to routine laboratory tests (complete blood count, metabolic panel, lipid profile, etc.), serum high-sensitivity cTnI (hs-cTnI) was measured using a chemiluminescent microparticle immunoassay (catalog no. 11291347, version 22.0) on the ADVIA Centaur XPT analyzer (Siemens Healthineers, Erlangen, Germany). The assay has a limit of detection of 1.6 pg/mL and a limit of quantitation of 2.5 pg/mL. The upper reference limits were 34.2 pg/mL in male participants and 15.6 pg/mL in female participants, according to the manufacturer’s specifications and validated laboratory reference standards. Analytical imprecision (coefficient of variation) was <10% at both decision limits.

### 2.3. Statistical Methods

Statistical analyses were conducted using IBM SPSS Statistics version 25.0 (IBM Corp., Chicago, IL, USA) and JASP 0.95.4 (2025, University of Amsterdam, The Netherlands). The normality of variables was assessed with the Shapiro–Wilk test. Continuous data are presented as means ± standard deviations, while categorical data are expressed as frequencies and percentages. Comparisons between groups were performed using the independent-samples *t* test for normally distributed variables and the Mann–Whitney U test for non-normally distributed variables. Differences between groups were further evaluated with the independent-samples *t* test, Chi-square test, or Fisher’s exact test, as appropriate. Associations between variables were evaluated using Pearson’s correlation coefficient. Univariate and multivariate logistic regression analyses were performed to identify predictors of mortality, with variable selection guided by sample size and predictor correlations to minimize multicollinearity. A *p*-value <0.05 was considered statistically significant, and *p*-values that could not be reported to three decimal places are shown as <0.001.

## 3. Results

### 3.1. Outcome-Based Comparison of Clinical and Radiological Parameters

In Table 2, we compare clinical and radiological features between patients who survived and those who died following sICH. The groups differed significantly in age, GCS scores, ICH volume, IVH, and ICH and NIHSS scores. Patients who died had lower GCS scores (*p* < 0.001), larger hematoma volumes (*p* < 0.001), a higher incidence of IVH (*p* < 0.001), and higher ICH and NIHSS scores (both *p* < 0.001) and were older (*p* = 0.015). In contrast, there was no significant difference in the infratentorial origin of hemorrhage between the two groups (*p* = 0.304).

### 3.2. Serum Concentrations of High-Sensitivity Cardiac Troponin I

**Figure 1:**
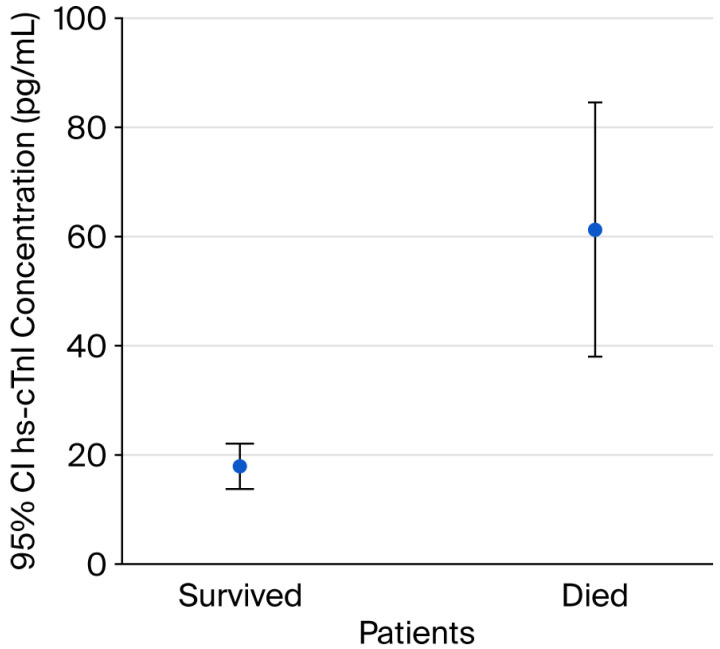
Serum levels of hs-cTnI in patients with sICH.

Figure 1 shows the mean serum concentrations of hs-cTnI (pg/mL) with 95% confidence intervals (CIs) in patients with sICH, comparing survivors and non-survivors. Patients who died had significantly higher serum hs-cTnI levels (61.3; 95% CI: 37.9–84.3) compared with survivors (17.8; 95% CI: 13.8–22) (*p* < 0.001).

### 3.3. Correlation Values

Table 3 presents the correlation between serum hs-cTnI levels and various clinical and radiological characteristics of sICH. A moderate positive correlation was observed between serum hs-cTnI levels and the total ICH score (r = 0.311, *p* = 0.002), as well as with individual parameters, including a lower GCS score (r = −0.262, *p* = 0.009), a larger ICH volume (r = 0.347, *p* < 0.001), and the presence of IVH (r = 0.342, *p* = 0.001). Positive correlations were also noted with infratentorial origin (r = 0.119, *p* = 0.240) and age (r = 0.031, *p* = 0.762), although these did not reach statistical significance. Furthermore, a significant positive correlation was observed between serum hs-cTnI concentrations and NIHSS (r = 0.381, *p* < 0.001).

### 3.4. Association of Clinical Scores and hs-cTnI with Mortality

Table 4 shows the results of the univariate logistic regression analysis assessing the association of ICH score, NIHSS, and serum hs-cTnI levels with mortality in patients with sICH. All three parameters were significantly associated with mortality: higher ICH scores (*p* < 0.001), higher NIHSS scores (*p* < 0.001), and elevated hs-cTnI concentrations (*p* = 0.001). Each 1-point increase in the ICH score was associated with an almost threefold increase in the odds of death, and each additional NIHSS point conferred approximately 44% higher odds of mortality. Moreover, each 1-unit increase in hs-cTnI levels was related to a 2.8% increase in the odds of death, underscoring the prognostic relevance of myocardial injury in patients with sICH.

Results from the multivariate logistic regression analysis are summarized in Table 5, in which the individual components of the ICH score were evaluated in relation to mortality. GCS score, ICH volume, and IVH emerged as significant factors associated with mortality, indicating that patients with lower GCS scores (*p* = 0.003), larger ICH volumes (*p* = 0.013), and the presence of IVH (*p* = 0.019) had a higher risk of death. Specifically, each 1-point decrease in the GCS score was associated with a 20% increase in the odds of death, while each 1 mL increase in ICH volume conferred a 1.7% increase in the odds of mortality. Moreover, the presence of IVH was associated with more than a fourfold increase in the odds of death compared with patients without IVH. Although infratentorial hematoma location and age showed a trend toward significance, they did not meet the conventional threshold to be considered predictors of mortality.

## 4. Discussion

As the second most common form of stroke, ICH carries markedly greater risks of morbidity and mortality compared with ischemic stroke and represents an important challenge for social and healthcare resources [4]. Despite diagnostic advances over the past few decades, no highly effective biomarker for predicting outcomes has been identified [31]. Numerous factors have been reported in the literature as potential predictors of poor outcomes and mortality in patients with sICH; however, the results often show inconsistency and lack comprehensive coverage [4,9,10,25,32]. Therefore, we aimed to systematically assess the association of the ICH score, NIHSS, and serum hs-cTnI levels with 30-day mortality, as well as the contribution of the individual ICH score components to mortality risk in patients with sICH.

In this study, we found that overall ICH and NIHSS scores were significantly higher in patients who did not survive sICH. In a Bangladeshi study, Ray SK et al. reported that elevated ICH scores were independent predictors of 30-day mortality and were strongly associated with unfavorable functional outcomes [33]. Similarly, other studies have shown that higher NIHSS scores are linked to a greater risk of death and worse functional recovery [12,32]. The results of our study indicated that patients who died from sICH were significantly older than those who survived. Although age showed a trend toward significance, it did not meet the conventional threshold to be considered an independent predictor of mortality. An international randomized controlled trial among 2839 patients with sICH (INTERACT2) showed that participants over 75 years of age had a fourfold higher risk of death or disability than those under 52 years [34]. A study conducted by Bahrami M et al. found that age over 65 years was significantly associated with increased risks of IVH and subarachnoid hemorrhage [35]. An aging population, coupled with the growing indications for the use of oral anticoagulant and antithrombotic agents, suggests that the number of anticoagulated patients will continue to increase, which might negatively impact the epidemiology of sICH [32,36]. Moreover, the incidence of hypertension, diabetes, and coronary heart disease, recognized as significant risk factors for worsening prognosis in patients with sICH, is higher in older individuals [35].

The patients in our study who died exhibited significantly lower GCS scores and greater hematoma volumes compared with those who survived. Furthermore, multiple logistic regression analysis identified GCS score and ICH volume as significant factors related to mortality. In Asian populations, low initial GCS scores and larger hematoma volumes have consistently been reported as independent predictors of poor outcomes [4,9]. A large multicenter case–control study in South Korea involving 1321 patients with sICH found that extensive white matter lesions were associated with lower GCS scores and higher mortality [37]. Ulger H et al. reported that patients with GCS values ≤9 had an increased risk of mortality and that hematoma volumes exceeding 44.16 mL were significant predictors of poor outcomes [31]. Initial ICH volumes greater than 30 mL have also been identified as significant predictors of mortality [9]. Conversely, some studies suggest that initial hematoma volume may be a better predictor of hematoma expansion than of mortality [38].

Our results showed that patients who died had significantly higher rates of IVH compared with those who survived. Moreover, the presence of IVH appeared to be a significant factor associated with mortality. The occurrence of IVH in patients with sICH has been established as a poor prognostic factor for hematoma expansion and fatal outcomes [9,32,38]. Neuroimaging features linked to an increase in-hospital mortality include the presence of IVH, brainstem hemorrhage, and signs of recurrent bleeding [39]. Recent studies have also demonstrated that perihematomal edema, a radiological indicator of secondary brain injury, is linked to poorer outcomes in patients with ICH, especially in those with basal ganglia hemorrhage [10,40]. Baseline factors associated with larger perihematomal edema include higher NIHSS scores, older age, lower GCS scores, larger ICH volumes, irregularly shaped hematomas, and higher glucose levels [41,42].

In our study, the groups did not differ significantly in terms of hemorrhage location. Infratentorial hemorrhage did not appear to be a significant factor related to fatal outcomes in patients with sICH, which contrasts with the findings of most other studies [15,29,43]. This discrepancy may, at least in part, be explained by the relatively small sample size of our cohort and the limited number of patients with infratentorial hemorrhage, resulting in reduced statistical power to detect such an association. Therefore, this negative finding should be interpreted with caution and does not exclude a potential prognostic impact of infratentorial hemorrhage, as suggested by larger multicenter studies.

Over the years, the uncertain and often poor prognosis of patients with sICH has prompted researchers to investigate the pathophysiological role of peripheral blood biomarkers in brain injury and their prognostic potential. In this context, serum concentrations of numerous biomarkers, including angiogenic and growth factors, inflammatory markers, coagulation parameters, and blood counts, have been evaluated in several studies [44,45]. Among these, cTnI is one of the most frequently measured biomarkers, as both observational studies and randomized trials have reported serious cardiac events in patients with stroke, including acute myocardial injury, acute coronary syndromes, left ventricular systolic and diastolic dysfunction, and arrhythmias [46,47].

Our study demonstrated that patients who died had significantly higher serum hs-cTnI concentrations compared with survivors. Furthermore, univariate logistic regression analysis showed that hs-cTnI was significantly associated with mortality in patients with sICH. Findings from the FAST trial, a multicenter prospective randomized study, suggested that patients with sICH and elevated serum hs-cTnI levels face increased risks of poor outcomes and higher mortality at 15 and 90 days compared with those without acute myocardial injury [26]. In addition, several studies have reported associations between elevated cTnI or cTnT levels and adverse functional outcomes, as well as increased mortality [27,46,48]. According to the American Heart Association and the American Stroke Association, patients with sICH should be assessed for coexisting myocardial injury and acute coronary ischemia using electrocardiography and hs-cTnI measurements [49]. Evidence from clinical studies and animal research indicates that cTn release is primarily driven by neurocardiogenic injury resulting from autonomic dysfunction. Excessive activation of the central autonomic network can lead to increased catecholamine release, which in turn may cause heightened adrenergic stimulation of the heart, resulting in coronary vasoconstriction, myocardial ischemia and necrosis, and arrhythmias. These cardiac changes associated with stroke have been collectively termed “stroke-heart syndrome” [22,47,50]. However, elevated hs-cTnI levels may reflect not only acute neurocardiac interactions following sICH but also underlying or concomitant cardiovascular conditions. Despite adjustment for documented comorbidities, the influence of unmeasured factors such as subclinical coronary artery disease, chronic kidney disease, and pulmonary embolism cannot be entirely excluded [21,22,26].

The results of our study showed a moderate positive correlation between serum hs-cTnI levels and NIHSS score, total ICH score, and its individual components, including a lower GCS score, larger hematoma volume, and the presence of IVH. Our results align with those of Qin G et al., who identified insular involvement, hematoma volume greater than 30 mL, and the presence of IVH as among the strongest predictors of elevated myocardial enzyme levels following the onset of sICH [51]. Elevated serum cTn levels were also significantly correlated with lower GCS and higher NIHSS scores [52]. Such associations may be explained by the crucial role of the insular cortical and subcortical regions in maintaining cardiac sympathetic output [53]. Furthermore, IVH expansion may trigger acute activation of the sympathetic nervous system, leading to myocardial injury and cardiac arrhythmia [52,54].

At the same time, some studies have not confirmed a clear link between elevated serum cTn levels and worse outcomes in patients with stroke [22,28,55]. Gulia A et al. suggested that elevated cTn levels may predict mortality in patients with acute ischemic stroke and subarachnoid hemorrhage, although this association was not observed in patients with sICH, highlighting the complexity of using cTn as a potential prognostic marker [23]. Overall, the literature presents conflicting evidence regarding the prognostic significance of cTn in acute stroke, which may be partly explained by the influence of factors unrelated to sICH on myocardial enzyme levels [51,56].

Some limitations of this study should be noted. First, the fact that it was a prospective observational cohort performed at a single center could restrict the generalizability of the results to other healthcare settings or populations. Second, the relatively small number of participants may have contributed to the lack of statistically significant associations between hs-cTnI levels and certain ICH characteristics, as well as to the failure of some individual components of the ICH score to emerge as significant predictors of mortality. Third, serum hs-cTnI was measured only once at admission, preventing assessment of dynamic changes over time and whether elevated levels were transient or persistent. Inclusion of serial hs-cTnI measurements along with baseline cardiac evaluation, such as electrocardiography, echocardiography, and assessment of prior cardiac history, would strengthen this research by clarifying whether elevated troponin levels reflect pre-existing cardiac conditions or acute changes related to sICH. The reliance on univariate analysis for hs-cTnI, without its inclusion in a dedicated multivariable model, limits the interpretation of hs-cTnI as an independent prognostic marker. Additionally, data on long-term functional outcomes, such as Modified Rankin Scale scores or mortality beyond 30 days, were not collected, limiting the ability to fully evaluate the potential prognostic relevance of hs-cTnI. Finally, although we adjusted for major clinical and radiological variables, the potential influence of unmeasured confounders, such as underlying coronary artery disease or variations in prehospital care, cannot be excluded. Despite these limitations, our study highlights the relationship of clinical scores and hs-cTnI with mortality in patients with sICH, providing a framework for future larger prospective studies.

## 5. Conclusions

Our results indicate that the ICH score, NIHSS, and serum hs-cTnI levels were significantly associated with 30-day mortality in patients with sICH. Individual components of the ICH score, including a lower GCS score, a larger hematoma volume, and the presence of IVH, emerged as independent factors related to mortality. These markers may aid clinicians in monitoring and managing patients effectively. Nevertheless, future multicenter studies with larger cohorts, inclusion of functional outcome measures, adjustment of hs-cTnI in a multivariable model alongside established predictors, and serial hs-cTnI measurements are warranted to validate these findings and further clarify their clinical utility in managing patients with sICH.

## Figures and Tables

**Table 1:** Components of the ICH score.

Component	Criteria	Points
GCS score	3–4	2
	5–12	1
	13–15	0
ICH volume	≥30 mL	1
	<30 mL	0
IVH	Present	1
	Absent	0
Infratentorial origin	Yes	1
	No	0
Age	≥80 years	1
	<80 years	0
Total Score Range		0–6

Estimated 30-day mortality risk: 0 points: 0%, 1 point: 13%, 2 points: 26%, 3 points: 72%, 4 points: 97%, 5–6 points: 100%. Abbreviations: GCS: Glasgow Coma Scale; ICH: intracerebral hemorrhage; IVH: intraventricular hemorrhage.

**Table 2:** Comparison of ICH score and its components and NIHSS score between patient groups.

Variable	Survived *n* = 65 (%)	Died *n* = 35 (%)	*p*
GCS score			<0.001 ^A^
3–4	2 (3.1)	11 (31.4)	
5–12	9 (13.8)	11 (31.4)	
13–15	54 (83.1)	13 (37.2)	
ICH volume (mL)			<0.001 ^B^
<30	47 (72.3)	9 (25.7)	
≥30	18 (27.7)	26 (74.3)	
IVH			<0.001 ^B^
Yes	15 (23.1)	26 (74.3)	
No	50 (76.9)	9 (25.7)	
Infratentorial origin			0.304 ^B^
Yes	8 (12.3)	7 (20.0)	
No	57 (87.7)	28 (80.0)	
Age (years)	71.35 ± 13.16	77.74 ± 10.34	0.015 ^C^
ICH score			<0.001 ^A^
0	17 (26.2)	2 (5.7)	
1	26 (40.0)	2 (5.7)	
2	11 (16.9)	5 (14.3)	
3	7 (10.8)	13 (37.1)	
4	4 (6.1)	6 (17.2)	
5	0 (0.0)	3 (8.6)	
6	0 (0.0)	4 (11.4)	
NIHSS	7.06 ± 5.06	23.11 ± 8.60	<0.001 ^C^

Abbreviations: *n* (%): number (percentage) of patients; GCS: Glasgow Coma Scale; ICH: intracerebral hemorrhage; IVH: intraventricular hemorrhage; NIHSS: National Institutes of Health Stroke Scale. Age is presented as mean ± standard deviation. ^A^ Fisher’s exact test; ^B^ Chi-square test; ^C^ *t* test for independent samples.

**Table 3:** Correlation of serum hs-cTnI levels with ICH score, its components, and NIHSS.

Variable	Serum Levels of hs-cTnI	
	r	*p*
ICH score	0.311	0.002
GCS score	−0.262	0.009
ICH volume	0.347	<0.001
IVH	0.342	0.001
Infratentorial origin	0.119	0.240
Age	0.031	0.762
NIHSS	0.381	<0.001

Abbreviations: hs-cTnI: high-sensitivity cardiac troponin I; GCS: Glasgow Coma Score; ICH: intracerebral hemorrhage; IVH: intraventricular hemorrhage; NIHSS: National Institutes of Health Stroke Scale.

**Table 4:** Association of ICH score, NIHSS score, and hs-cTnI levels with mortality.

Model	Variable	B	Exp (B)	95% CI	*p*
1	ICH score	1.017	2.764	1.829–4.178	<0.001
2	NIHSS	0.367	1.443	1.241–1.679	<0.001
3	Serum hs-cTnI levels	0.027	1.028	1.012–1.044	0.001

Abbreviations: ICH: intracerebral hemorrhage; NIHSS: National Institutes of Health Stroke Scale; hs-cTnI: high-sensitivity cardiac troponin I; CI: confidence interval.

**Table 5:** Relationship between ICH score components and mortality.

Variable	B	Exp (B)	95% CI	*p*
GCS score	−0.227	0.797	0.686–0.925	0.003
ICH volume	0.017	1.017	1.004–1.031	0.013
IVH	1.507	4.514	1.277–15.958	0.019
Infratentorial origin	−0.183	0.833	0.139–4.996	0.841
Age	0.044	1.045	0.989–1.104	0.116

Abbreviations: ICH: intracerebral hemorrhage; CI: confidence interval; GCS: Glasgow Coma Score; IVH: intraventricular hemorrhage.

## Data Availability

The original contributions presented in this study are included in the article. Further inquiries can be directed to the corresponding authors.

## Abbreviations

The following abbreviations are used in this manuscript:

CI	Confidence interval
cTn	Cardiac troponin
GCS	Glasgow Coma Scale
hs-cTnI	High-sensitivity cardiac troponin I
ICH	Intracerebral hemorrhage
IVH	Intraventricular hemorrhage
NIHSS	National Institutes of Health Stroke Scale
sICH	Spontaneous intracerebral hemorrhage
UHC	University Hospital Center
